# Cyclooxygenase-2 promotes tumor growth and suppresses tumor immunity

**DOI:** 10.1186/s12935-015-0260-7

**Published:** 2015-11-05

**Authors:** Bing Liu, Liyan Qu, Shigui Yan

**Affiliations:** Department of Orthopedics, 2nd Affiliated Hospital, School of Medicine, Zhejiang University, #88 Jie Fang Road, 310009 Hangzhou, Zhejiang People’s Republic of China; Clinical Laboratory Centre, 2nd Affiliated Hospital, School of Medicine, Zhejiang University, #88 Jie Fang Road, 310009 Hangzhou, Zhejiang People’s Republic of China; Clinical Laboratory Centre, Binjiang Hospital of Hangzhou, Hangzhou, Zhejiang People’s Republic of China

**Keywords:** COX-2, COX-inhibitors, EP, Innate immunity, Adaptive immunity

## Abstract

Cyclooxygenase-2 (COX-2), an inducible form of the enzyme that catalyzes the first step in the synthesis of prostanoids, is associated with inflammatory diseases and carcinogenesis, which is suspected to promote angiogenesis and tissue invasion of tumors and resistance to apoptosis. Meanwhile, COX-2 contributes to immune evasion and resistance to cancer immunotherapy, which plays a crucial role in the innate and adaptive immune response. The activity of COX-2-PGE2-EP signal pathway can suppress Dendritic cells (DCs), natural killer (NK), T cells, type-1 immunity excluding type-2 immunity which promote tumor immune evasion. COX-2 and the prostaglandin cascade play important roles in the “inflammogenesis of cancer”. In addition, COX-inhibitors can inhibit tumor immune evasion. Therefore, we can exert the COX-inhibitors to facilitate the patients to benefit from addition of COX-inhibitors to standard cytotoxic therapy.

## Background

Human malignancies generally arise as the culmination of a multistep process that involves various somatic gene alterations. Therefore, we can exert the drug to affect over-expressed or low-expressed genes and achieve the therapies of human malignancies. It was learned that COX-2 is overexpressed in most solid tumors such as colorectal, liver, pancreatic, breast as well as lung cancer [1–6]. Both non-selective non-steroidal anti-inflammatory drugs (NSAIDs) and selective COX-2 inhibitors can inhibit proliferation, tumors invasiveness and angiogenesis, and at the same time overcome apoptosis and drug resistance as well as suppress of immune responses. Immune responses include innate immunity and adaptive immunity. Tumor-associated immune responses can be generalized to type 1, in which Th1 lymphocytes and M1-polarized macrophages limit tumor progression, and type 2, in which Th2 lymphocytes and M2 macrophages favor immune escape and disease progression [7]. Natural Killer (NK) cells are a subset of lymphocytes that participate in innate immunity. Dendritic cells bridge innate and adaptive immunity and participate in both responses. Tumor-associated macrophages (TAMs) have emerged as promising target for anti-cancer immunotherapy. MDSC block adaptive immunity. Cytotoxic T cells directed against antigens that are endogenously expressed and presented by cancer cells are critically involved in antigen-specific cancer immunotherapy.

Meanwhile, COX-2 contributes to immune evasion and resistance to cancer immunotherapy. The activity of COX-2 -PGE2-EPs signal pathway can suppress Dendritic cells (DCs), natural killer (NK), T cells, type-1 immunity, but promote type-2 immunity, which promote tumor immune evasion. COX-2 inhibitors may have off-target effects on immune cells and can counterbalance their activity as enhancers of susceptibility to immune elimination. So, COX-2 may serve as predictive biomarker and as therapeutic target for modulation of immune resistance in cancer.

## Cyclooxygenase

The cyclooxygenase (COX) isoenzymes, known as prostaglandin (PG) rate-limiting synthase, catalyze the metabolism of arachidonic acid (AA) to PGs. Finally, a series of biologically active prostaglandins (PGD2, PGE2, PGF2α, and PGI2) and thromboxane A2 (TXA2) are formed. There are three isoforms of the enzyme that have been identified: COX-1, COX-2, and COX-3 [8]. Considered as a “housekeeping enzyme”, COX-1 is constitutively expressed in human cells. COX-3, an alternate splice variant of COX-1, is most abundant in the canine cerebral cortex. COX-2 is an inducible enzyme and is associated with inflammatory diseases and carcinogenesis, which is suspected to promote angiogenesis and tissue invasion of tumors [9, 10] and resistance to apoptosis [8, 11]. Moreover, COX-2-dependent prostaglandin release can suppress antigen presentation and immune activation in cancer [12]. Therefore, COX-2 and the prostaglandin cascade play important roles in the “inflammogenesis of cancer”.

## COX-2: PGE_2_-prostaglandin E receptors signal pathway

Arachidonic acid (AA) is transformed into unstable intermediate PGG2, which is promptly converted into PGH2 by cyclooxygenases (COXs) and finally into five primary prostaglandins (PGD_2_, PGE_2_, PGF_2α_, PGI_2_, and TXA_2_) by cell-specific synthases. The actions of these prostanoid ligands are mediated by their engagement of specific cell-surface G-protein-coupled receptors designated EP1–4 for PGE2 [13]. It is widely accepted that alterations of cyclooxygenase-2 (COX-2) expression as well as its massive enzymatic product PGE2 play a key role in influencing the development of cancer because their level is found markedly elevated in tissues of cancer [1–6].

Both COX-1 and COX-2 are capable of converting AA into prostaglandins. However, they exhibit preferentially in synthesizing prostaglandins [14]. It has been demonstrated that PGE_2_ and PGI_2_ are mainly derived from the COX-2 pathway [15]. PGE2 is generated from PGH2 by cytosolic PGE2 synthase (cPGES) and membrane-bound microsomal PGE2 synthase-1 and -2 (mPGEs-1 and -2). Once PGE2 is synthesized, it diffuses immediately and activates its specific membrane receptors (EP1–4). EP1 receptors couple with the Gq-phospholipase C(PLC)-inositol trisphosphate (IP3) pathway and its activation results in the release of intracellular Ca2+. EP2 and EP4 receptors couple with the Gs-adenylyl cyclase (AC)-cAMP-protein kinase A (PKA) pathway. EP3 couples with a pertussis toxin-sensitive Gi protein to inhibit AC resulting in a decrease in cAMP [15].

Chell et al. [16] report that compared with normal colonic epithelium, protein expression will increased in colorectal cancers (100 %) as well as adenomas (36 %) when using immunohistochemistry in vivo EP4 receptor. PGE2 signaling through the EP4 receptor has previously been associated with colorectal tumorigenesis. Fujino H reported that EP2 receptor mediated activation of Tcf transcriptional activity is primarily through a cAMP/protein kinase A (PKA) dependent mechanism; whereas, EP4 receptor mediated activation occurs primarily through a phosphatidylinositol 3-kinase (PI3K) dependent pathway [17]. Recently, they have found that PGE2 stimulation of EP4 receptors activates an additional PI3K-dependent pathway leading to the phosphorylation of the extracellular signal-regulated kinases (ERKs), followed by induction of the functional expression of early growth response factor 1 (EGR-1) [18, 19]. ERKs phosphorylation and induction of EGR-1 expression was unique for EP4 receptors and was not observed in cells that expressed EP2 receptors [18, 19]. Cyclin D1, a key regulator of cell cycle progression, is under the control of EGR-1 through a PI3K- and ERK-dependent pathway [20]. EP4-dependent activation of PI3K/Akt signaling has been reported to stimulate the proliferation and motility of colorectal cancer cells [21]. Therefore, it concerns a possible role for EP4 receptors in cancer.

The role which EP2 receptors played in cancer is still controversial. Sonoshita et al. [22] showed that homozygous deletion of the gene encoding EP2 would cause the decreases in number and size of intestinal polyps in ApcΔ716 mice (a mouse model for human familial adenomatous polyposis). Homozygous gene knockout for other PGE2 receptors, EP1 or EP3, did not affect the intestinal polyp formation in ApcΔ716 mice. Fujino et al. [17] suggested that the increased expression of COX-2 via EP2 receptors and an increased expression of PGE2 synthase via EP4 receptors could explain the increased biosynthesis of PGE2 known to occur in colon cancer.

## COX-2: PGE_2_-prostaglandin E receptors suppress innate and adaptive immunity

COX-2: PGE2-prostaglandin E receptors contributes to immune evasion and resistance to cancer immunotherapy (Table 1), which have been reported to augment pro-tumorigenic type 2 lymphocyte [23]. COX-2: PGE2 has been considered as a major product and modulator of activated macrophages [24] for a long time. Tumor-associated macrophages (TAMs) represent a major subpopulation of tumor infiltrating immune cells, which has emerged as a promising target for anti-cancer immunotherapy. Hindering macrophage polarization towards a pro-tumor M2 phenotype, or better still reprogramming the M2 like TAMs towards M1 subtype is being considered a beneficial anti-cancer strategy. COX-2/PGE2 Pathway Promotes M2 Macrophage Differentiation [25, 26]. Macrophage-mediated immune suppression is correlated with increased CD4+ CD25+ regulatory T cell infiltration and reduced CD8+ cytotoxic T cell function.

**Table 1:** The role of COX-2 as an oncogene or suppression of tumor immunity

Oncogene	Promotes angiogenesis and tissue invasion of tumors [7, 8], resistance to apoptosis [9, 10]
	COX-2-PGE2-Prostaglandin E Receptors signal pathway [12]
	PI3K-dependent pathway [16]
	Extracellular signal-regulated kinases (ERKs) [17]
	Early growth response factor 1 (EGR-1) [18]
Innate immunity	
Macrophages	Augment pro-tumorigenic type 2 lymphocyte [22]
	Promotes M2 macrophage differentiation [24, 25]
Natural Killer (NK) cells	Inhibits the potential of NK cells to migrate, exert cytotoxic effects, and secrete interferonγ [26]
	Inhibits major NK receptors (NKR): NKG2D, CD16 and natural cytotoxicity receptors (NCR: NKp30, NKp44, NKp46) [27]
Dendritic cells	A key immunomodulator of DC biology [28]
	Reduces the maturation of DCs and their expression of MHC class II molecules [29]
	The production of endogenous IL-10 adaptive immunity
Adaptive immunity	
B and T cells	Inhibits proliferation of B and T lymphocytes [31]
	Bluntes the interferon-gamma release of antigen-specific T cells and increased the expression of interleukin-4 and indoleamine
	2,3-dioxygenase by tumor cells [32]
γδ T cells	Inhibits γδ T cell receptors TCR Vγ9Vδ2, NKG2D, CD16 [27]
Tregs	Induces Tregs [34]
	Promotes CD4+ and CD8+ T cells differentiation in Tregs [33]
	Inhibits effector T cells in a COX-2-dependent manner [35, 36]
MDSC	COX-2 would maintain elevated MDSC levels [40]

Natural Killer (NK) cells are a subset of lymphocytes that participate in innate immunity. NK cells express all PGE2 EP receptors, and tumor-derived PGE2 represents a major barrier to the success of NK cell mediated killing. PGE2 inhibits the potential of NK cells to migrate, exert cytotoxic effects, and secrete interferonγ [27]. Martinet et al. [28] also demonstrate that the major NK receptors (NKR): NKG2D, CD16 and natural cytotoxicity receptors (NCR: NKp30, NKp44, NKp46) are all inhibited by PGE2. The ability of PGE2 to inhibit NK cells is by acting on EP2 and EP4 receptors. They also report that a recently described EP4 antagonist, frondoside A, inhibits breast tumor metastasis in an NK-dependent manner and protects IFNγ production by NK cells from PGE2 mediated suppression.

Dendritic cells could bridge the innate and adaptive immunity and participate in both responses. COX-2/PGE2 has become a key immunomodulator of DC biology [29], which can exert inhibitory activity and reduce the maturation of DCs and their expression of MHC class II molecules, ability to present antigen and activate T cells [30]. Meanwhile, prostaglandin E2 enhances the production of endogenous IL-10, which down-regulates dendritic cell functions. PGE2 modulates dendritic cell function via EP2 and EP4 receptor subtypes. It is suggested that targeting PGE2 EP2/EP4 receptor signaling may be a powerful mechanism for modulating DC activity [31].

Over-expression of COX-2 can initiate and promote carcinogenesis by inhibiting proliferation of B and T lymphocytes, particularly natural killer T cells, thus limiting antineoplastic activity (immunosuppression) [32]. Göbel et al. [33] have identified COX-2—PGE2 as resistance factor against the cytotoxicity induced by activated, antigen-specific T cells. COX-2 expression blunted the interferon-gamma release of antigen-specific T cells exposed to their respective cellular targets, and increased the expression of interleukin-4 and indoleamine 2,3-dioxygenase by tumor cells.

It has been reported that γδ T cell receptors TCR Vγ9Vδ2, NKG2D and CD16 are all inhibited by PGE2 [28]. PGE2 also contributes to inhibit directly the proliferation and effector functions of CD4+ and CD8+ T cells and to promote their differentiation in regulatory T cells [34]. COX-2/PGE(2) induces Tregs, and Treg cells support the cancer-mediated immune suppression. The extent of COX-2 expression was associated significantly with Treg prevalence (P = 0.004) and Treg intratumoral localization (P = 0.005) [35]. Several studies have demonstrated that Treg cells contribute to immunosuppression in cancer and inhibit effector T cells in a COX-2-dependent manner [36, 37]. The suppressive activity of T regulatory cells is driven by expression of the forkhead/winged helix transcription factor (FOXP3) gene [38, 39].

MDSC are present in many cancers, which block adaptive immunity by inhibiting the activation of CD4+ and CD8+ T cells [40] and innate immunity by inhibiting natural killer cells [41].Once a tumor is established, tumor cell production of COX2 would maintain elevated MDSC levels, further blocking tumor immunity and allowing the malignant cells to proliferate without interference from the host’s immune system. This provides a rationale for therapeutic targeting of COX-2 expression and stress-induced prostaglandin synthesis to boost immune surveillance and immunotherapy of cancer.

Above all, COX-2—PGE2- EP2/4 signal pathway plays an important part in the development of cancer; therefore, the EP2/4 receptors may represent an important target for cancer prevention and treatment.

## COX-2 inhibitors inhibit immune evasion

Conventional non-steroidal anti-inflammatory drugs (NSAIDs) and COX-2 selective inhibitors have been demonstrated to overcome tumor immune evasion. It has been suggested that COX-inhibitors may sensitize type 1 immune responses via inhibiting M2 macrophage, T regulatory cells, MDSC; enhancing DC, NK, cytotoxic T-lymphocyte functions [42]. Use of selective COX-2 inhibitors could result in a significant risk reduction for each type of cancer (71 % for breast cancer, 55 % for prostate cancer, 70 % for colon cancer, and 79 % for lung cancer) and an overall 68 % risk reduction for all four cancers. This investigation demonstrates that COX-2 blocking agents have strong potential for the chemoprevention of cancers [32].

PGE2 inhibits production of the inflammatory chemokines CCL3 and CCL4, which is mediated through EP2 and EP4 receptors, preventing excess accumulation of activated immune cells [43]. Further analysis revealed that COX inhibitors and EP4 receptor antagonism contribute to NK functions critical to the control of metastatic disease. In vivo, NK cells were necessary for the therapeutic effects of COX inhibitors. Further, mammary tumor cells treated with COX inhibitors were more sensitive to NK mediated lysis. Moreover, expression of inhibitory ligands for NK cells were decreased and stimulatory ligands were increased by treatment with COX inhibitors or EP4 receptor antagonists. These studies suggest that EP4, expressed on malignant cells, is a potential therapeutic target [26, 44, 45].

COX-2 inhibition would cause the loss of the M2 macrophage characteristics of TAMs and may assist prevention of breast cancer metastasis. Chen et al. [46] suggest that inhibition of myeloid cell COX-2 can potentiate CTL-mediated tumor cytotoxicity and may provide a novel therapeutic approach in breast cancer therapy. They found that the specific COX-2 inhibitor, inhibited human M2 macrophage differentiation, as determined by decreased CD14 and CD163 expressions and increased TNFγ production. At the same time, COX-2 inhibition alters the phenotype of tumor-associated macrophages from M2 to M1 in ApcMin/+ mouse polyps.

Meanwhile, COX-2 inhibition potentiates macrophage’s inflammatory cytokine responses but reduces IL-10 secretion thus might skew overall tumor microenvironment to favor Th1 immune responses which was confirmed by reduced regulatory T cells as well as myeloid derived suppressor cells in etodolac fed mice tumor mass [45]. COX-2 KO mice resulted from disruption of M2-like TAM function, and thereby enhancing T cell survival and immune surveillance [46]. PGE2 seems to influence the antigen-specific cellular responses of both T cells and APCs because reversal of inhibition was seen by pretreatment of both with COX-2 inhibitor. COX-2 inhibitor attenuates Treg cell activity and Foxp3 expression in tumor-infiltrating lymphocytes and enhances antitumor responses [47, 48], which still delays primary tumor growth and reduces MDSC accumulation [49].

Meta-analysis of independent estimates from 72 studies provides no evidence that the selective COX-2 inhibitor will influence the relative risk of cardiovascular disease (composite relative risk = 0.98, 95 % CI = 0.88–1.10) [50]. Meanwhile, the mechanisms of COX-2 inhibitors regulate the tumor immune evasion still needs further research.

## Conclusion

In this review, we have tried to encompass the role of COX-2 in the regulation of tumor immune evasion. The activity of COX-2 is suspected to promote angiogenesis, tissue invasion of tumors and resistance to apoptosis and chemotherapy. The activity of COX-2-PGE2-Prostaglandin E Receptors signal pathway can suppress Dendritic cells (DCs), natural killer (NK), T cells, type-1 immunity, but promote type-2 immunity, which promote tumor immune evasion. COX-2 and the prostaglandin cascade play an important part in the “inflammogenesis of cancer”. In addition, COX-inhibitors can inhibit tumor immune evasion. Therefore, we can exert the COX-inhibitors benefit from addition of COX-inhibitors to standard cytotoxic therapy, which can overcome tumor immune evasion.
